# Risk Factors for Trauma-Induced Coagulopathy- and Transfusion-Associated Multiple Organ Failure in Severely Injured Trauma Patients

**DOI:** 10.3389/fmed.2015.00024

**Published:** 2015-04-24

**Authors:** Kirsten Balvers, Mathijs R. Wirtz, Susan van Dieren, J. Carel Goslings, Nicole P. Juffermans

**Affiliations:** ^1^Trauma Unit, Department of Surgery, Academic Medical Center, Amsterdam, Netherlands; ^2^Department of Intensive Care, Academic Medical Center, Amsterdam, Netherlands; ^3^Clinical Research Unit, Academic Medical Center, Amsterdam, Netherlands

**Keywords:** trauma, multiple organ failure, transfusion, coagulopathy, resuscitation

## Abstract

**Background:**

Both trauma-induced coagulopathy (TIC) and transfusion strategies influence early outcome in hemorrhagic trauma patients. Their impact on late outcome is less well characterized. This study systematically reviews risk factors for TIC- and transfusion-associated multiple organ failure (MOF) in severely injured trauma patients.

**Materials and methods:**

A systematic search was conducted in PubMed and Embase. Studies published from 1986 to 2013 on adult trauma patients with an injury severity score ≥16, investigating TIC or transfusion strategies with MOF as primary or secondary outcome, were eligible for inclusion. Results of the included studies were evaluated with meta-analyses of pooled data.

**Results:**

In total, 50 studies were included with a total sample size of 63,586 patients. Due to heterogeneity of the study populations and outcome measures, results from 7 studies allowed for pooling of data. Risk factors for TIC-associated MOF were hypocoagulopathy, hemorrhagic shock, activated protein C, increased histone levels, and increased levels of markers of fibrinolysis on admission. After at least 24 h after admission, the occurrence of thromboembolic events was associated with MOF. Risk factors for transfusion-associated MOF were the administration of fluids and red blood cell units within 24 h post-injury, the age of red blood cells (>14 days) and a ratio of FFP:RBC ≥ 1:1 (OR 1.11, 95% CI 1.04–1.19).

**Conclusion:**

Risk factors for TIC-associated MOF in severely injured trauma patients are early hypocoagulopathy and hemorrhagic shock, while a hypercoagulable state with the occurrence of thromboembolic events later in the course of trauma predisposes to MOF. Risk factors for transfusion-associated MOF include administration of crystalloids and red blood cells and a prolonged storage time of red blood cells. Future prospective studies investigating TIC- and transfusion-associated risk factors on late outcome are required.

## Introduction

Despite advances in trauma care, multiple organ failure (MOF) still remains one of the leading causes of late mortality (occurring after more than 3 days) in trauma patients (1, 2). The incidence of MOF in severely injured trauma patients ranges from 15% up until 40% (3–6), with an associated mortality rate that varies between 24% (3) and 51% (6). Even though MOF-related mortality has been shown to decrease over the last decades (2, 6), mortality is still 10 times higher in patients with MOF compared to patients without MOF (4, 5).

Over the last decade, trauma-induced coagulopathy (TIC) is increasingly recognized to contribute to adverse early outcome in trauma patients (7–13). In recognition of that, transfusion strategies have changed toward more and earlier administration of plasma. This has led to a shift in the ratio of RBC:FFP to 1:1. Furthermore, fluid resuscitation with crystalloids has evolved from aggressive therapy to a minimal amount of crystalloid administration. More and earlier administration of plasma, combined with a restriction of crystalloid administration, has showed to reduce early mortality (14–16). However, the impact of both TIC and changing transfusion strategies on the occurrence of MOF has not been systematically reviewed before. Therefore, the aim of this study was to summarize risk factors for TIC- and transfusion-associated MOF in severely injured trauma patients.

## Materials and Methods

The present study was reported according to the PRISMA guidelines (preferred reporting items for systematic reviews and meta-analyses) (17).

### Study selection

An electronic search was conducted in PubMed and Embase for articles published from 1986 to 2013. In addition, we searched for ongoing trials on www.controlled-trials.com and www.clinicaltrials.gov.

The following subject headings and free text words were used: (“Blood Coagulation Disorders”[Mesh] OR “Blood Coagulation”[Mesh] OR Coagulation[tiab] OR coagulopathy[tiab] OR “Fibrinolysis”[Mesh] OR Fibrinolysis[tiab] OR hypofibrinolysis[tiab] OR hyperfibrinolysis[tiab]) OR (“Blood Transfusion”[Mesh] OR Transfusion[tiab] OR “Transfusion Medicine”[Mesh] OR “Erythrocyte Transfusion”[Mesh] OR “Blood Component Transfusion”[Mesh]) AND (“Multiple Organ Failure”[Mesh] OR multiple organ failure*[tiab] OR MOF[tiab] OR (infection*[tiab] AND trauma[tiab])) AND (“Multiple Trauma”[Mesh] OR multiple trauma[tiab] OR “Wounds and Injuries”[Mesh] OR “Injury Severity Score”[Mesh] OR Injury Severity Score[tiab] OR ISS[tiab] (Table S2 in Supplementary Material).

Target population were trauma patients who suffered blunt or penetrating trauma, with a mean injury severity score (ISS) of ≥16 and an age of ≥16 years. Randomized controlled trials (RCTs) and observational studies investigating TIC or transfusion strategies with MOF as primary or secondary outcome were eligible for inclusion. Studies, which focused on patients with isolated traumatic brain injury or burn injury, were excluded. Both prospective and retrospective studies were included. Reviews, correspondences, case reports, expert opinions, and editorials were excluded. The search was conducted by two independent researchers (Kirsten Balvers and Mathijs R. Wirtz). Any discrepancies in the included studies were resolved by discussion between the reviewers. If necessary, an independent third reviewer was consulted. Only articles defining MOF according to the definition of the Denver (18), Marshall (18, 19), or SOFA (20) score were included in this review. A Denver score of more than 3 and a Marshall score of more than 5, both for at least two consecutive days, were used to define MOF. Furthermore, MOF according to the SOFA score was defined as the simultaneous failure of two or more organ systems. Organ failure was defined as a total of more than two points in a single organ. Language was limited to English, Dutch, or German. We reviewed the bibliographies of the eligible studies for citations of additional suitable studies.

### Data synthesis

Primary outcomes were risk factors for TIC- and transfusion-associated MOF. Since most of the studies in this field are observational studies, we performed a quality assessment according to the Newcastle-Ottawa Scale (21). Characteristics of the studies examined included comparability of the study groups, methods used to select study participants and determination of outcome variables. The quality of selection of patients in the included studies was rated as good if they included severely injured trauma patients and the control group was drawn from the same community as the exposed cohort. The assessment of comparability of the studies was based on the design and/or analysis used in the studies. Quality of outcome variables was determined by follow-up period and <10% of patients lost-to-follow-up. The Cochrane Collaboration’s tool for assessing the risk of bias was used to assess the quality of RCTs (22). This tool was used to evaluate RCTs on seven specific domains (sequence generation, allocation concealment, blinding of participants and personnel, blinding of outcome assessment, incomplete outcome data, selective reporting, and other sources of bias). If the results of studies were contradicting, the quality assessment was used to grade conclusions.

Review Manager (RevMan 5, The Nordic Cochrane Centre) was used to combine findings of studies in a meta-analysis. Studies were pooled if homogeneity was considered by assessing study population, intervention, and outcome. RevMan was used to determine homogeneity by the inverse variance method for a random or fixed effects model. If homogeneity was not obtained studies were excluded from meta-analysis. Heterogeneity was expressed by *I*^2^. An *I*^2^ of >75% was considered as substantial heterogeneity. Meta-analysis was performed on observational studies and RCTs, in which data from observational studies and RCTs were not combined in the same meta-analysis. For the outcome of interest, risk ratios and 95% confidence intervals were used.

## Results

**Figure 1:**
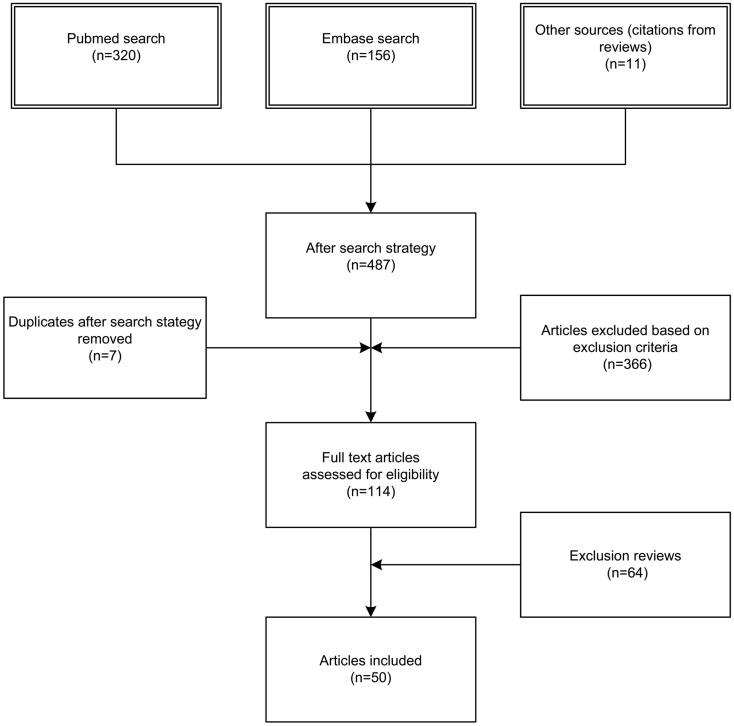
**The process of selecting studies suitable for inclusion**.

We identified 476 articles (PubMed 320, Embase 156) meeting the inclusion criteria. Of these, seven duplicates were removed. Reviewing of the bibliographies resulted in 11 additional articles. The full texts of 114 articles were assessed for eligibility. An additional 64 reviews were excluded, bringing the total on 50 included articles with a total sample size of 63,586 patients (Figure 1). Of the 50 included studies, 46 studies were observational cohort studies and 4 were RCTs. The observational studies included 15 retrospective and 31 prospective studies. Sample size in these studies varied between 19 and 20,288 patients with a median of 384 (IQR 135–1217) patients. Two studies included a heterogeneous population of intensive care patients, all other studies were restricted to trauma patients. The score of the included studies on the Newcastle-Ottawa scale ranged from 6 to 8 with a median of 7. The score of the Cochrane Collaboration’s tool for assessing the risk of bias ranged from 8 to 9 (Tables 1 and 2; Table S1 in Supplementary Material).

**Table 1:** **Description of included studies; risk factors for TIC-associated MOF**.

Reference	Design	Origin	Patients	*N*	Groups	Risk factors for MOF	Quality
Nuytinck et al. (23)	Prospective	Europe	Trauma patients	71	ARDS/MOF	Plasma elastase level	7/9
					Non-ARDS/MOF	Complement activation	
Wudel et al. (24)	Retrospective	USA	Trauma patients	92	Survivors	No difference	7/9
					Non-survivors	
Sigurddson et al. (25)	Prospective	Asia	Critically ill patients	21	Hemorrhagic shock	Platelet activity and intestinal platelet sequestration	7/9
					controls	
Waydhas et al. (26)	Prospective	Europe	Trauma patients	133	MOF	No difference in coagulopathy	7/9
					Non-MOF	Platelet count <180,000/μL	
Gando et al. (27)	Prospective	Japan	Trauma patients	58	DIC	DIC	6/9
					Non-DIC	
Gando et al. (28)	Prospective	Japan	Trauma patients	47	DIC	Thrombomodulin level	6/9
					Non-DIC	DIC	
Sauaia et al. (29)	Retrospective	USA	Trauma patients	411	MOF	Colloid administration	9/9
					Non-MOF	Lower platelet count	
						Longer prothrombin time	
Gando et al. (30)	Prospective	Japan	Trauma patients	136	SIRS for ≤2 days	SIRS ≥3 days	6/9
					SIRS for ≥3 days	Platelet counts	
					Non-SIRS	DIC	
Raeburn et al. (31)	Retrospective	USA	Trauma patients	77	Abdominal compartment syndrome (ACS)	No difference	7/9
Newell et al. (32)	Retrospective	USA	Trauma patients	1751	Normal	VTE	7/9
					Overweight	
					Obese	
					Morbidly obese	
Maegele et al. (33)	Retrospective	Europe	Trauma patients	8724	Coagulopathy	Coagulopathy	7/9
					Non-coagulopathy	
Paffrath et al. (34)	Retrospective	Europe	Trauma patients	7937	VTE	VTE	7/9
					Non-VTE	
Nydam et al. (35)	Retrospective	USA	Trauma patients	1415	Thrombocytopenia	Thrombocytopenia	8/9
					Non-thrombocytopenia	
Brown et al. (36)	Prospective	USA	Trauma patients	1877	Acute traumatic coagulopathy	Activation of protein C	7/9
					Non-acute traumatic coagulopathy	Acute coagulopathy	
					Male versus female	Transfusion requirements	
Kutcher et al. (37)	Prospective	USA	Trauma patients	132	High histone levels	High histone level	7/9
					Low histone levels	
Cohen et al. (38)	Prospective	USA	Trauma patients	203	–	Higher levels of activated protein C upon admission	8/9
Cole et al. (39)	Prospective	Europe	Trauma patients	158	Infection	PC depletion of PC and raised PAP levels	7/9
					Non-infection	
Trentzsch et al. (40)	Retrospective	Europe	Trauma patients	20,288	Male	No difference in coagulopathy	8/9
					Female	

**Table 2:** **Description of included studies; risk factors for transfusion-associated MOF**.

Reference	Design	Origin	Patients	*N*	Groups	Risk factors for MOF	Quality
Sauaia et al. (41)	Retrospective	USA	Trauma patients	394	MOF	>6 RBCs	8/9
					Non-MOF	
Lehmann et al. (42)	Retrospective	Europe	Trauma patients	1112	MOF	RBC administration	8/9
					Non-MOF	Crystalloids	
Moore et al. (43)	Prospective	USA	Trauma patients	513	MOF	Blood transfusion products	8/9
					Non-MOF	
Waydhas et al. (44)	RCT	Europe	Trauma patients	40	ATIII placebo	Placebo	8/10
Sauaia et al. (29)	Retrospective	USA	Trauma patients	411	MOF	Colloid administration	9/9
					Non-MOF	Lower platelet count	
						Longer prothrombin time	
Zallen et al. (45)	Prospective	USA	Trauma patients	63	MOF	Number of and a	8/9
					Non-MOF	Age of blood units	
Cryer et al. (46)	Prospective	USA	Trauma patients	105	MOF	>6 RBC units	8/9
					Non-MOF	
Ciesla et al. (6)	Prospective	USA	Trauma patients	1344	MOF	Blood products	8/9
					Non-MOF	Transfusion of >6 RBCs	
Frink et al. (47)	Prospective	Europe	Trauma patients	143	MOF	Transfusion	7/9
					Non-MOF	
Bulger et al. (48)	RCT	USA	Trauma patients	209	Hypertonic fluids	No difference	9/10
					Ringer solution	
Sperry et al. (50)	Prospective	USA	Trauma patients	415	FFP:PRBC ≥ 1:1.50	A high FFP:PRBC ratio patients	8/9
					FFP:PRBC ≤ 1:1.51	
Maegele et al. (51)	Retrospective	Europe	Trauma patients	713	RBC:FFP > 1.1	RBC: FFP 0⋅9–1⋅1 (1:1)	8/9
					RBC:FFP 0.9–1.1	
					RBC:FFP <0.9	
Holcomb et al. (52)	Retrospective	USA	Trauma patients	467	Low plasma:RBC < 1:2	No difference	8/9
					high plasma:RBC ratio >1:2	
					Low platelet:RBC < 1:2	
					high platelet:RBC ratio >1:2	
Jastrow et al. (53)	Prospective	USA	Trauma patients	48	MOF	Transfusion FFPs and RBCs	7/9
					Non-MOF	
Englehart et al. (54)	Prospective	USA	Trauma patients	1036	RBCs leukoreduced	No difference	6/9
					RBCs not leukoreduced	
Dewar et al. (55)	Retrospective	USA	Trauma patients	504	MOF	No difference	7/9
					Non-MOF	
Mahambrey et al. (56)	Retrospective	Canada	Trauma patients	260	–	RBC administration	7/9
Watson et al. (57)	Prospective	USA	Trauma patients	1175	High plasma transfusion	FFP and cryoprecipitate	9/9
					Low plasma transfusion	
Boffard et al. (58)	RCT	Africa	Trauma patients	301	rVIIa Placebo	rVII group lower incidence MOF although not significant	8/10
Cotton et al. (59)	Prospective	USA	Trauma patients	266	Pre-massive transfusion protocol	Blood product administration	7/9
					Massive transfusion protocol	
Hauser et al. (60)	RCT	World wide	Trauma patients	573	FVIIa Placebo	A trend is observed toward decreased MOF in rFVIIa group	9/10
Paffrath et al. (34)	Retrospective	Europe	Trauma patients	7937	VTE	VTE	7/9
					Non-VTE	
Brattstrom et al. (5)	Prospective	Europe	Trauma patients	164	–	>10 RBC units	8/9
Johnson et al. (61)	Retrospective	USA	Trauma patients	1440	MOF	RBC administration	8/9
					Non-MOF	
Nienaber et al. (62)	Retrospective	Europe	Trauma patients	36	FFP Coagulation factor concentrates	PCC treatment associated with reduction of MOF	7/9
Perkins et al. (63)	Retrospective	USA	Trauma patients	369	Fresh whole blood	No difference	7/9
					Apheresis platelets	
Wafaisade et al. (64)	Retrospective	Europe	Trauma patients	1362	FFP:RBC < 1:1	No difference	7/9
					FFP:RBC 1:1	
					FFP:RBC > 1:1	
Hussmann et al. (65)	Retrospective	Europe	Trauma patients	375	<1000 ml	Crystalloids <1000 ml	7/9
					1000–2000 ml	
					2001–3000 ml	
					>3000 ml	
Brakenridge et al. (66)	Prospective	USA	Trauma patients	1366	–	>10 RBC units	7/9
Borgman et al. (67)	Retrospective	Europe	Trauma patients	2474	High FFP:RBC > 1:2	No difference	8/9
					Low FFP:RBC < 1:2	
Brown et al. (36)	Prospective	USA	Trauma patients	1877	Acute traumatic coagulopathy	Crystalloid, RBC, and FFP administration	7/9
					Non-acute traumatic coagulopathy	
Innerhofer et al. (68)	Prospective	Europe	Trauma patients	144	Fibrinogen and/or prothrombin complex concentrate alone	FFP administration	8/9
					Additionally FFP	
Minei et al. (69)	Prospective	USA	Trauma patients	916	MOF	FFP administration	9/9
					Non-MOF	Crystalloid administration	
Neal et al. (70)	Prospective	USA	Trauma patients	452	Crystalloid:RBC ratio	Crystalloid:RBC ratio >1.5:1	9/9
Duchesne et al. (71)	Retrospective	USA	Trauma patients	188	Hypertonic solution	Isotonic solution	7/9
					Isotonic solution	

### Risk factors for TIC-associated MOF

**Figure 2:**
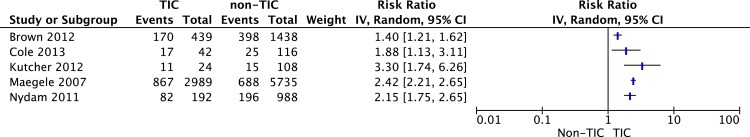
**The impact of TIC on the development of MOF**. Studies have reported an association between TIC and the incidence of MOF; however, pooling of data was not possible due to substantial heterogeneity.

Eighteen studies reported the effect of TIC on the development of MOF in trauma patients (Table 1). The presence of hypocoagulopathy on admission to the emergency department (ED) was an independent risk factor for MOF (26, 30, 33, 35–39); however, studies could not be pooled due to substantial heterogeneity (*I*^2^ = 90%, Figure 2). Hypocoagulopathy was defined by prolongation of coagulation parameters including PTT, INR, and APTT and a decreased platelet count (26). Four studies reported a decreased platelet count as an independent risk factor (23, 26, 41, 72). Of note, hypocoagulopathy was rare in patients without persisting shock (73). Other risk factors for TIC-associated MOF were activation of protein C, increased levels of fibrinolytic markers (27, 36–39), and increased levels of extracellular histones (37). Of note, these risk factors were reported in small study numbers.

Taken together, after trauma, damaged endothelial cells and extracellular histones activate protein C, which inhibits factor Va and VIIa and leads to hyperfibrinolysis due to the consumption of plasminogen activator inhibitor, with subsequent hypocoagulopathy (37, 74–76).

Later in the course of events following trauma, patients tend to develop a hypercoagulopathy as reported in 5 studies with a total of 5581 patients. In these studies, an association between thromboembolic events, including disseminated intravascular coagulation (DIC) and venous thromboembolism (VTE), and MOF was reported (27, 28, 30, 32, 34). Pooling of data in a meta-analysis was not possible due to differences in outcome measures.

### Risk factors for transfusion-associated MOF

We found 36 studies reporting an association between transfusion and the development of MOF in trauma patients (Table 2).

#### Fluids

Six studies investigated the effect of the administration of crystalloids on MOF in trauma patients. The majority of studies reported crystalloid administration within the first 24 h post-injury as a risk factor for the development of MOF (36, 42, 67, 69, 70). Another study showed a trend toward a lower incidence of MOF in patients who were administered <1000 ml of fluids prior to arrival at the hospital. Two studies did not find a relation between fluids and MOF (53, 65). However, these two studies did not adjust for confounders. Pooling of data could not be performed due to difference in outcome measures. However, it is likely that crystalloid administration is an independent risk factor for MOF given that the studies, which adjusted for confounders found an association between the administration of crystalloids and MOF.

#### Blood products

The effect of the amount of RBCs administered on the development of MOF in trauma patients was reported in 14 studies (5, 6, 36, 41–43, 45, 46, 55, 56, 61, 66). There seems to be a dose-dependent association between MOF and transfusion, as a significant linear trend was found between the number of RBCs transfused and the incidence of MOF (43, 49). In addition, most studies reported an increased risk for MOF after administration of more than six units; however, studies could not be pooled due to differences in outcomes measures. Besides the amount of RBCs administrated, the age of red blood cells of >14 days was found as an independent risk factor in four studies. Storage of RBCs for over 14 days was reported to increase the risk of MOF with an OR of 1.16 (95% CI 1.02–1.32; *P* = 0.03). The OR increased to 1.22 (95% CI 1.06–1.41; *P* = 0.006) when the RBC units were older than 21 days (45).

**Figure 3:**
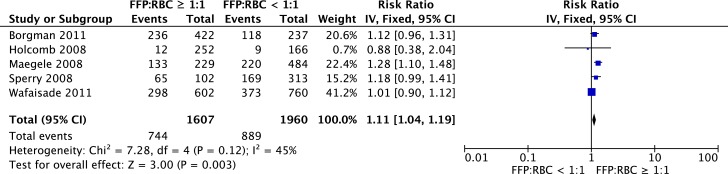
**Meta-analysis: the impact of a high FFP:RBC ratio (≥1:1) versus a low FFP:RBC ratio (<1:1) on the development of MOF**. A significant association between a high FFP:RBC ratio and the incidence of MOF is observed (*P* = 0.003).

Eight studies investigated the effect of FFPs on the development of MOF. Two studies observed a relation between the administration of FFPs and MOF (57, 69). Other studies reported merely a trend or results were not adjusted for confounders (33, 36, 42, 50, 52, 53). When data of five observational studies were pooled for meta-analysis, there was a significant association between a high FFP:RBC ratio of ≥1:1 and MOF (RR 1.11, 95% CI 1.04–1.19, Figure 3). Of note, studies were limited in design. The effect of platelets on the development of MOF was investigated in five studies. No significant association between platelet administration and MOF was reported in these studies (52, 53, 57, 61, 63).

#### Procoagulant agents

**Figure 4:**
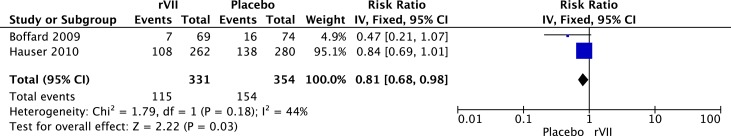
**Meta-analysis: the effect of administration of rVII on the development of MOF**. A significant lower incidence of MOF was observed in patients with rVII compared to patients with placebo (*P* = 0.03).

Five studies reported on the relation between MOF and the use of procoagulant agents in patients with severe hemorrhage. In an RCT with 573 patients, recombinant factor VII (rVII) significantly reduced transfusion requirements in both blunt and penetrating trauma patients and showed a trend toward a lower MOF rate in blunt trauma patients (60). Another RCT showed a lower incidence of MOF in patients treated with rVII, although these results were not significant (58). Pooling of data from these two RCTs suggested a lower incidence of MOF in patients treated with rVII compared to placebo (RR 0.81, 95% CI 0.68–0.98, Figure 4).

The early and high-dose administration of antithrombin (AT) significantly reduced duration of MOF, but did not reduce the incidence of MOF (44). Of note, there was no significant difference in safety profile, including thromboembolic events, between the groups. Two studies reported that prothrombin complex concentrate (PCC) administration resulted in decreased transfusion requirements with an associated significant lower frequency of MOF in severely injured trauma patients (62, 77).

In summary, the limitedly available data suggest that procoagulant agents do not contribute to a higher incidence of thromboembolic events and subsequently MOF in severe trauma patients. In fact, these agents are associated with reduced transfusion requirements and a reduced incidence of MOF.

## Discussion

Risk factors for TIC-associated MOF in severely injured trauma patients are early hypocoagulopathy, whereas later in the course after admission, the occurrence of thromboembolic events was associated with MOF. Risk factors for transfusion-associated MOF were the administration of fluids and red blood cell units, the age of red blood cells and an FFP:RBC ratio ≥1:1. Risk factors are summarized in Table 3.

**Table 3:** **Risk factors for TIC- and transfusion-associated MOF**.

**Trauma-induced coagulopathy**
Hypocoagulpathy on emergency department (26, 30, 33, 35–39)
High activated protein C levels on emergency department (36, 38, 39)
High extracellular histones levels on emergency department (37)
Depleted PC levels during hospital stay (38)
Thromboembolic events, e.g., DIC and DVT during hospital stay (76, 78)
**Transfusion strategy**
Transfusion of RBCs, FFPs, and crystalloids within first 24 h post-injury (6, 36, 41, 43, 45, 65)
Age RBC >2 weeks (45)

**Figure 5:**
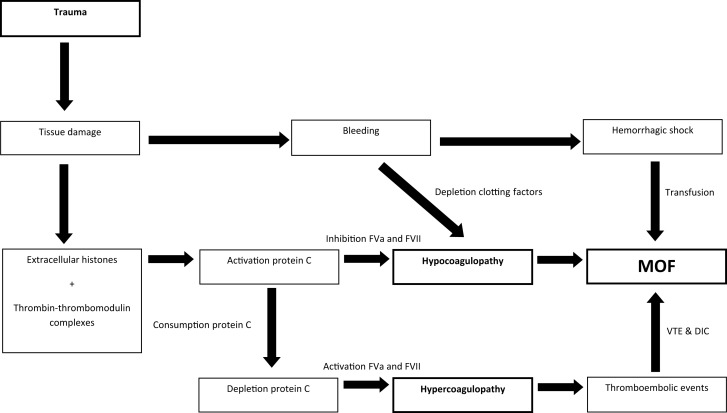
**Linking hypo- and hypercoagulopathy in the development of MOF in trauma patients; a hypothesis**.

Hemorrhagic shock and early presence of hypocoagulopathy are risk factors for MOF in trauma patients. Subsequently, after at least 24-h after admission, thromboembolic events were reported as risk factors. Thereby, the coagulation profile associated with MOF seems to change over time. In an effort to reconcile these findings, we hypothesize that patients can transfer from a hypocoagulable state on admission toward a hypercoagulable state later during the hospital stay, which may predispose to MOF. Immediately after tissue injury, thrombomodulin complexes and extracellular histones activate protein C, which leads to hypocoagulopathy due to the inhibition of FVa and FVII and hyperfibrinolysis (28, 37, 74, 75). Activation of protein C results in utilization of protein C. If protein C levels are consumed and patients do not recover their protein C levels, inhibition of FVa and VII will not occur, causing a hypercoagulable state. This may be followed by the formation of vascular thrombi leading to cell damage in organs and eventually MOF (Figure 5). Further studies are required to confirm this hypothesis.

Risk factors for transfusion-associated MOF are administration of crystalloids, transfusion of RBCs, the age of RBCs >14 days and an FFP:RBC ratio ≥1:1. When transfusion of fluids and blood products is inevitable a limited amount of fluid and blood products is recommended. We found that a high FFP:RBC ratio is an independent risk factor for MOF. However, since transfusion with a low FFP:RBC ratio of <1:1 is associated with a higher mortality due to bleeding (50, 51, 79, 80), clear recommendations on the FFP:RBC ratio, with the aim to limit MOF cannot be made. In particular, due to the different scoring systems used to define MOF in the meta-analysis. Further studies on risks and benefits of blood product ratios are warranted. A possible explanation for the association between the administration of RBCs in trauma patients and MOF may be storage time. However, the use of fresh blood only is probably not feasible in exsanguinating trauma patients. Furthermore, limited data in this study suggest that procoagulant agents do not contribute to a higher incidence of thromboembolic events and subsequently MOF in severely injured trauma patients. In fact, they seem to reduce the risk of MOF, which is most likely related to a decrease in transfusion requirements. Whether the addition of procoagulant agents may decrease transfusion requirements and subsequently the development of MOF remains to be determined.

### Limitations

There are several limitations to this review. The included studies have a considerable risk of bias related to design and methodology and several studies did not adjust for confounders. Also, there was a relevant heterogeneity as data were presented as mean or median, as frequencies and percentages, and as odds ratios with 95% confidence intervals. This hampered pooling of data in the meta-analysis. Pooling of data was feasible in 7 out of the 50 included studies. Additionally, we have used the Newcastle-Ottawa Scale to assess the quality of observational studies. Previous studies reported a low reliability of the scale due to differences in assessment and low agreement between reviewers, which is a limitation of the scale and subsequently of this study (81, 82). However, despite these limitations, the Cochrane Collaboration recommends the Newcastle-Ottawa scale as the most useful tool for assessing the risk of bias in non-RCTs (83). Furthermore, there is a lack of a uniform definition of MOF. The use of different scores of MOF hampers interpretation of the results of the meta-analyses and therefore no firm conclusions can be drawn. Additional studies are required to confirm the results of this study.

## Conclusion

Identifying patients at high risk for MOF may guide the need for monitoring of organ failure and may provide avoidance of therapy, which can aggravate organ failure. Early hypocoagulopathy and shock are risk factors for TIC-associated MOF in severely injured trauma patients. Later in the course of trauma, a hypercoagulable state with the occurrence of thromboembolic events predisposes to MOF. Risk factors for transfusion-associated MOF include the administration of crystalloids and red blood cells and a prolonged storage time of red blood cells. However, pooling of data was hampered by heterogeneity of the study populations and outcome measures. Future prospective studies investigating TIC- and transfusion-associated risk factors on late outcome are required.

## Conflict of Interest Statement

The authors declare that the research was conducted in the absence of any commercial or financial relationships that could be construed as a potential conflict of interest.

## Supplementary Material

The Supplementary Material for this article can be found online at http://journal.frontiersin.org/article/10.3389/fmed.2015.00024/abstract

## Abbreviations

ED, emergency department; FFP, fresh frozen plasma; ISS, injury severity score; MOF, multiple organ failure; RBC, red blood cell; RCT, randomized controlled trial; rVII, recombinant factor VII; TIC, trauma-induced coagulopathy.

## References

[1] Sauaia A Moore FA Moore EE Moser KS Brennan R Read RA Epidemiology of trauma deaths: a reassessment. J Trauma (1995) 38(2):185–93. 10.1097/00005373-199502000-00006 7869433

[2] Soreide K Kruger AJ Vardal AL Ellingsen CL Soreide E Lossius HM. Epidemiology and contemporary patterns of trauma deaths: changing place, similar pace, older face. World J Surg (2007) 31(11):2092–103. 10.1007/s00268-007-9226-9 17899256

[3] Dewar DC Tarrant SM King KL Balogh ZJ. Changes in the epidemiology and prediction of multiple-organ failure after injury. J Trauma Acute Care Surg (2013) 74(3):774–9. 10.1097/TA.0b013e31827a6e69 23425734

[4] Nast-Kolb D Aufmkolk M Rucholtz S Obertacke U Waydhas C. Multiple organ failure still a major cause of morbidity but not mortality in blunt multiple trauma. J Trauma (2001) 51(5):835–41. 10.1097/00005373-200111000-00003 11706328

[5] Brattstrom O Granath F Rossi P Oldner A. Early predictors of morbidity and mortality in trauma patients treated in the intensive care unit. Acta Anaesthesiol Scand (2010) 54(8):1007–17. 10.1111/j.1399-6576.2010.02266.x 20626360

[6] Ciesla DJ Moore EE Johnson JL Burch JM Cothren CC Sauaia A. A 12-year prospective study of postinjury multiple organ failure: has anything changed? Arch Surg (2005) 140(5):432–8. 10.1001/archsurg.140.5.432 15897438

[7] Dunn EL Moore EE Breslich DJ Galloway WB Acidosis-induced coagulopathy. Surg Forum (1979) 30:471–3. 538668

[8] Schochl H Frietsch T Pavelka M Jambor C. Hyperfibrinolysis after major trauma: differential diagnosis of lysis patterns and prognostic value of thrombelastometry. J Trauma (2009) 67(1):125–31. 10.1097/TA.0b013e31818b2483 19590321

[9] Johansson PI Stensballe J Rasmussen LS Ostrowski SR. A high admission syndecan-1 level, a marker of endothelial glycocalyx degradation, is associated with inflammation, protein C depletion, fibrinolysis, and increased mortality in trauma patients. Ann Surg (2011) 254(2):194–200. 10.1097/SLA.0b013e318226113d 21772125

[10] MacLeod JB Lynn M McKenney MG Cohn SM Murtha M. Early coagulopathy predicts mortality in trauma. J Trauma (2003) 55(1):39–44. 10.1097/01.TA.0000075338.21177.EF 12855879

[11] Hess JR Lindell AL Stansbury LG Dutton RP Scalea TM. The prevalence of abnormal results of conventional coagulation tests on admission to a trauma center. Transfusion (2009) 49(1):34–9. 10.1111/j.1537-2995.2008.01944.x 18954393

[12] Brohi K Singh J Heron M Coats T. Acute traumatic coagulopathy. J Trauma (2003) 54(6):1127–30. 10.1097/01.TA.0000069184.82147.06 12813333

[13] Plotkin AJ Wade CE Jenkins DH Smith KA Noe JC Park MS A reduction in clot formation rate and strength assessed by thrombelastography is indicative of transfusion requirements in patients with penetrating injuries. J Trauma (2008) 64(2 Suppl):S64–8. 10.1097/TA.0b013e318160772d 18376174

[14] del Junco DJ Holcomb JB Fox EE Brasel KJ Phelan HA Bulger EM Resuscitate early with plasma and platelets or balance blood products gradually: findings from the PROMMTT study. J Trauma Acute Care Surg (2013) 75(1 Suppl 1):S24–30. 10.1097/TA.0b013e31828fa3b9 23778507 PMC3744122

[15] Brown LM Aro SO Cohen MJ Holcomb JB Wade CE Brasel KJ A high fresh frozen plasma: packed red blood cell transfusion ratio decreases mortality in all massively transfused trauma patients regardless of admission international normalized ratio. J Trauma (2011) 71(2 Suppl 3):S358–63. 10.1097/TA.0b013e318227f152 21814104 PMC4374732

[16] Morrison CA Carrick MM Norman MA Scott BG Welsh FJ Tsai P Hypotensive resuscitation strategy reduces transfusion requirements and severe postoperative coagulopathy in trauma patients with hemorrhagic shock: preliminary results of a randomized controlled trial. J Trauma (2011) 70(3):652–63. 10.1097/TA.0b013e31820e77ea 21610356

[17] Moher D Liberati A Tetzlaff J Altman DG Preferred reporting items for systematic reviews and meta-analyses: the PRISMA statement. BMJ (2009) 339:b2535 10.1136/bmj.b2535 19622551 PMC2714657

[18] Sauaia A Moore EE Johnson JL Ciesla DJ Biffl WL Banerjee A. Validation of postinjury multiple organ failure scores. Shock (2009) 31(5):438–47. 10.1097/SHK.0b013e31818ba4c6 18838942 PMC4324473

[19] Grotz M von GM Stalp M Kaufmann U Hildebrand F Pape HC. [Scoring multiple organ failure after severe trauma. Comparison of the Goris, Marshall and Moore scores]. Chirurg (2001) 72(6):723–30. 10.1007/s001040170130 11469095

[20] Antonelli M Moreno R Vincent JL Sprung CL Mendoca A Passariello M Application of SOFA score to trauma patients. Sequential organ failure assessment. Intensive Care Med (1999) 25(4):389–94. 10.1007/s001340050863 10342513

[21] Wells G Shea B O’Connel J The Newcastle-Ottawa Scale (NOS) for Assessing the Quality of Nonrandomised Studies in Meta-Analysis (2011). Available from: http://www.ohri.ca/programs/clinical_epidemiology/oxford.asp

[22] Higgins JPT Altman DG Chapter 8: assessing risk of bias in included studies. In: Higgins J Green S, editors. The Cochrane Collaboration, 5.0.1 ed Chichester: John Wiley & Sons, Ltd (2008).

[23] Nuytinck JK Goris JA Redl H Schlag G van Munster PJ. Posttraumatic complications and inflammatory mediators. Arch Surg (1986) 121(8):886–90. 10.1001/archsurg.1986.01400080028004 3488050

[24] Wudel JH Morris JA Jr Yates K Wilson A Bass SM. Massive transfusion: outcome in blunt trauma patients. J Trauma (1991) 31(1):1–7. 10.1097/00005373-199101000-00001 1986111

[25] Sigurdsson GH Christenson JT El-Rakshy MB Sadek S. Intestinal platelet trapping after traumatic and septic shock. An early sign of sepsis and multiorgan failure in critically ill patients? Crit Care Med (1992) 20:458–67. 10.1097/00003246-199204000-00005 1559357

[26] Waydhas C Nast-Kolb D Kick M Zettl R Wiesholler J Trupka A [Operation planning of secondary interventions after polytrauma]. Unfallchirurg (1994) 97(5):244–9. 8052860

[27] Gando S Nakanishi Y Tedo I. Cytokines and plasminogen activator inhibitor-1 in posttrauma disseminated intravascular coagulation: relationship to multiple organ dysfunction syndrome. Crit Care Med (1995) 23(11):1835–42. 10.1097/00003246-199511000-00009 7587259

[28] Gando S Nakanishi Y Kameue T Nanzaki S. Soluble thrombomodulin increases in patients with disseminated intravascular coagulation and in those with multiple organ dysfunction syndrome after trauma: role of neutrophil elastase. J Trauma (1995) 39(4):660–4. 10.1097/00005373-199510000-00007 7473950

[29] Sauaia A Moore FA Moore EE Norris JM Lezotte DC Hamman RF. Multiple organ failure can be predicted as early as 12 hours after injury. J Trauma (1998) 45(2):291–301. 10.1097/00005373-199808000-00014 9715186

[30] Gando S Nanzaki S Kemmotsu O. Disseminated intravascular coagulation and sustained systemic inflammatory response syndrome predict organ dysfunctions after trauma: application of clinical decision analysis. Ann Surg (1999) 229(1):121–7. 10.1097/00000658-199901000-00016 9923809 PMC1191617

[31] Raeburn CD Moore EE Biffl WL Johnson JL Meldrum DR Offner PJ The abdominal compartment syndrome is a morbid complication of postinjury damage control surgery. Am J Surg (2001) 182:542–6. 10.1016/S0002-9610(01)00821-2 11839314

[32] Newell MA Bard MR Goettler CE Toschlog EA Schenarts PJ Sagraves SG Body mass index and outcomes in critically injured blunt trauma patients: weighing the impact. J Am Coll Surg (2007) 204(5):1056–61. 10.1016/j.jamcollsurg.2006.12.042 17481540

[33] Maegele M Lefering R Yucel N Tjardes T Rixen D Paffrath T Early coagulopathy in multiple injury: an analysis from the German trauma registry on 8724 patients. Injury (2007) 38(3):298–304. 10.1016/j.injury.2006.10.003 17214989

[34] Paffrath T Wafaisade A Lefering R Simanski C Bouillon B Spanholtz T Venous thromboembolism after severe trauma: incidence, risk factors and outcome. Injury (2010) 41(1):97–101. 10.1016/j.injury.2009.06.010 19608183

[35] Nydam TL Kashuk JL Moore EE Johnson JL Burlew CC Biffl WL Refractory postinjury thrombocytopenia is associated with multiple organ failure and adverse outcomes. J Trauma (2011) 70(2):401–6. 10.1097/TA.0b013e31820b5c85 21307741

[36] Brown JB Cohen MJ Minei JP Maier RV West MA Billiar TR Characterization of acute coagulopathy and sexual dimorphism after injury: females and coagulopathy just do not mix. J Trauma Acute Care Surg (2012) 73(6):1395–400. 10.1097/TA.0b013e31825b9f05 23064602 PMC3540988

[37] Kutcher ME Xu J Vilardi RF Ho C Esmon CT Cohen MJ. Extracellular histone release in response to traumatic injury: implications for a compensatory role of activated protein C. J Trauma Acute Care Surg (2012) 73(6):1389–94. 10.1097/TA.0b013e318270d595 23188230 PMC3577065

[38] Cohen MJ Call M Nelson M Calfee CS Esmon CT Brohi K Critical role of activated protein C in early coagulopathy and later organ failure, infection and death in trauma patients. Ann Surg (2012) 255(2):379–85. 10.1097/SLA.0b013e318235d9e6 22133894 PMC3549308

[39] Cole E Davenport R De’Ath H Manson J Brockamp T Brohi K. Coagulation system changes associated with susceptibility to infection in trauma patients. J Trauma Acute Care Surg (2013) 74(1):51–7. 10.1097/TA.0b013e3182788b0f 23271077

[40] Trentzsch H Nienaber U Behnke M Lefering R Piltz S. Female sex protects from organ failure and sepsis after major trauma haemorrhage. Injury (2014) 45(Suppl 3):S20–8. 10.1016/j.injury.2014.08.013 25284229

[41] Sauaia A Moore FA Moore EE Haenel JB Read RA Lezotte DC. Early predictors of postinjury multiple organ failure. Arch Surg (1994) 129(1):39–45. 10.1001/archsurg.1994.01420250051006 8279939

[42] Lehmann U Grotz M Regel G Rudolph S Tscherne H. [Does initial management of polytrauma patients have an effect on the development of multiple organ failure? Evaluation of preclinical and clinical data of 1,112 polytrauma patients]. Unfallchirurg (1995) 98(8):442–6. 7570038

[43] Moore FA Moore EE Sauaia A. Blood transfusion. An independent risk factor for postinjury multiple organ failure. Arch Surg (1997) 132(6):620–4. 10.1001/archsurg.1997.01430300062013 9197854

[44] Waydhas C Nast-Kolb D Gippner-Steppert C Trupka A Pfundstein C Schweiberer L High-dose antithrombin III treatment of severely injured patients: results of a prospective study. J Trauma (1998) 45(5):931–40. 10.1097/00005373-199811000-00015 9820705

[45] Zallen G Offner PJ Moore EE Blackwell J Ciesla DJ Gabriel J Age of transfused blood is an independent risk factor for postinjury multiple organ failure. Am J Surg (1999) 178(6):570–2. 10.1016/S0002-9610(99)00239-1 10670874

[46] Cryer HG Leong K McArthur DL Demetriades D Bongard FS Fleming AW Multiple organ failure: by the time you predict it, it’s already there. J Trauma (1999) 46(4):597–604. 10.1097/00005373-199904000-00007 10217221

[47] Frink M Pape HC van Griensven M Krettek C Chaudry IH Hildebrand F. Influence of sex and age on mods and cytokines after multiple injuries. Shock (2007) 27:151–6. 10.1097/01.shk.0000239767.64786 17224789

[48] Bulger EM Cuschieri J Warner K Maier RV. Hypertonic resuscitation modulates the inflammatory response in patients with traumatic hemorrhagic shock. Ann Surg (2007) 245(4):635–41. 10.1097/01.sla.0000251367.44890.ae 17414614 PMC1877049

[49] Sperry JL Nathens AB Frankel HL Vanek SL Moore EE Maier RV Characterization of the gender dimorphism after injury and hemorrhagic shock: are hormonal differences responsible? Crit Care Med (2008) 36(6):1838–45. 10.1097/CCM.0b013e3181760c14 18496363

[50] Sperry JL Ochoa JB Gunn SR Alarcon LH Minei JP Cuschieri J An FFP:PRBC transfusion ratio >/=1:1.5 is associated with a lower risk of mortality after massive transfusion. J Trauma (2008) 65(5):986–93. 10.1097/TA.0b013e3181878028 19001962

[51] Maegele M Lefering R Paffrath T Tjardes T Simanski C Bouillon B. Red-blood-cell to plasma ratios transfused during massive transfusion are associated with mortality in severe multiple injury: a retrospective analysis from the trauma registry of the Deutsche Gesellschaft fur Unfallchirurgie. Vox Sang (2008) 95(2):112–9. 10.1111/j.1423-0410.2008.01074.x 18557827

[52] Holcomb JB Wade CE Michalek JE Chisholm GB Zarzabal LA Schreiber MA Increased plasma and platelet to red blood cell ratios improves outcome in 466 massively transfused civilian trauma patients. Ann Surg (2008) 248(3):447–58. 10.1097/SLA.0b013e318185a9ad 18791365

[53] Jastrow KM III Gonzalez EA McGuire MF Suliburk JW Kozar RA Iyengar S Early cytokine production risk stratifies trauma patients for multiple organ failure. J Am Coll Surg (2009) 209(3):320–31. 10.1016/j.jamcollsurg.2009.05.002 19717036

[54] Englehart MS Cho SD Morris MS Gee AC Riha G Underwood SJ Use of leukoreduced blood does not reduce infection, organ failure, or mortality following trauma. World J Surg (2009) 33(8):1626–32. 10.1007/s00268-009-0076-5 19452207 PMC7101844

[55] Dewar DC Mackay P Balogh Z. Epidemiology of post-injury multiple organ failure in an Australian trauma system. ANZ J Surg (2009) 79(6):431–6. 10.1111/j.1445-2197.2009.04968.x 19566865

[56] Mahambrey TD Fowler RA Pinto R Smith TS Callum JL Pisani NS Early massive transfusion in trauma patients: Canadian single-centre retrospective cohort study. Can J Anaesth (2009) 56(10):740–50. 10.1007/s12630-009-9151-5 19641979

[57] Watson GA Sperry JL Rosengart MR Minei JP Harbrecht BG Moore EE Fresh frozen plasma is independently associated with a higher risk of multiple organ failure and acute respiratory distress syndrome. J Trauma (2009) 67(2):221–7 10.1097/TA.0b013e3181ad5957 19667872

[58] Boffard KD Choong PI Kluger Y Riou B Rizoli SB Rossaint R The treatment of bleeding is to stop the bleeding! Treatment of trauma-related hemorrhage. Transfusion (2009) 49(Suppl 5):240S–7S 10.1111/j.1537-2995.2008.01987.x 19954486

[59] Cotton BA Au BK Nunez TC Gunter OL Robertson AM Young PP. Predefined massive transfusion protocols are associated with a reduction in organ failure and postinjury complications. J Trauma (2009) 66(1):41–8; discussion 48–9. 10.1097/TA.0b013e31819313bb 19131804

[60] Hauser CJ Boffard K Dutton R Bernard GR Croce MA Holcomb JB Results of the CONTROL trial: efficacy and safety of recombinant activated factor VII in the management of refractory traumatic hemorrhage. J Trauma (2010) 69(3):489–500. 10.1097/TA.0b013e3181edf36e 20838118

[61] Johnson JL Moore EE Kashuk JL Banerjee A Cothren CC Biffl WL Effect of blood products transfusion on the development of postinjury multiple organ failure. Arch Surg (2010) 145(10):973–7. 10.1001/archsurg.2010.216 20956766

[62] Nienaber U Innerhofer P Westermann I Schochl H Attal R Breitkopf R The impact of fresh frozen plasma vs coagulation factor concentrates on morbidity and mortality in trauma-associated haemorrhage and massive transfusion. Injury (2011) 42(7):697–701. 10.1016/j.injury.2010.12.015 21392760

[63] Perkins JG Cap AP Spinella PC Shorr AF Beekley AC Grathwohl KW Comparison of platelet transfusion as fresh whole blood versus apheresis platelets for massively transfused combat trauma patients (CME). Transfusion (2011) 51(2):242–52. 10.1111/j.1537-2995.2010.02818.x 20796254

[64] Wafaisade A Maegele M Lefering R Braun M Peiniger S Neugebauer E High plasma to red blood cell ratios are associated with lower mortality rates in patients receiving multiple transfusion (4 ≤red blood cell units <10) during acute trauma resuscitation. J Trauma (2011) 70(1):81–8; discussion 88–9. 10.1097/TA.0b013e3182032e0b 21217485

[65] Hussmann B Taeger G Lefering R Waydhas C Nast-Kolb D Ruchholtz S [Lethality and outcome in multiple injured patients after severe abdominal and pelvic trauma. Influence of preclinical volume replacement – an analysis of 604 patients from the trauma registry of the DGU]. Unfallchirurg (2011) 114(8):705–12. 10.1007/s00113-010-1842-4 21152886

[66] Brakenridge SC Phelan HA Henley SS Golden RM Kashner TM Eastman AE Early blood product and crystalloid volume resuscitation: risk association with multiple organ dysfunction after severe blunt traumatic injury. J Trauma (2011) 71(2):299–305. 10.1097/TA.0b013e318224d328 21825930 PMC3716363

[67] Borgman MA Spinella PC Holcomb JB Blackbourne LH Wade CE Lefering R The effect of FFP:RBC ratio on morbidity and mortality in trauma patients based on transfusion prediction score. Vox Sang (2011) 101(1):44–54. 10.1111/j.1423-0410.2011.01466.x 21438884 PMC3155292

[68] Innerhofer P Westermann I Tauber H Breitkopf R Fries D Kastenberger T The exclusive use of coagulation factor concentrates enables reversal of coagulopathy and decreases transfusion rates in patients with major blunt trauma. Injury (2013) 44:209–16. 10.1016/j.injury.2012.08.047 23000050

[69] Minei JP Cuschieri J Sperry J Moore EE West MA Harbrecht BG The changing pattern and implications of multiple organ failure after blunt injury with hemorrhagic shock. Crit Care Med (2012) 40(4):1129–35. 10.1097/CCM.0b013e3182376e9f 22020243 PMC3343366

[70] Neal MD Hoffman MK Cuschieri J Minei JP Maier RV Harbrecht BG Crystalloid to packed red blood cell transfusion ratio in the massively transfused patient: when a little goes a long way. J Trauma Acute Care Surg (2012) 72(4):892–8. 10.1097/TA.0b013e31823d84a7 22491601 PMC3347772

[71] Duchesne JC Simms E Guidry C Duke M Beeson E McSwain NE Damage control immunoregulation: is there a role for low-volume hypertonic saline resuscitation in patients managed with damage control surgery? Am Surg (2012) 78(9):962–8. 22964205

[72] Pape HC van GM Rice J Gansslen A Hildebrand F Zech S Major secondary surgery in blunt trauma patients and perioperative cytokine liberation: determination of the clinical relevance of biochemical markers. J Trauma (2001) 50(6):989–1000. 10.1097/00005373-200106000-00004 11426112

[73] Frith D Goslings JC Gaarder C Maegele M Cohen MJ Allard S Definition and drivers of acute traumatic coagulopathy: clinical and experimental investigations. J Thromb Haemost (2010) 8(9):1919–25. 10.1111/j.1538-7836.2010.03945.x 20553376

[74] Floccard B Rugeri L Faure A Saint DM Boyle EM Peguet O Early coagulopathy in trauma patients: an on-scene and hospital admission study. Injury (2012) 43(1):26–32. 10.1016/j.injury.2010.11.003 21112053

[75] Raza I Davenport R Rourke C Platton S Manson J Spoors C The incidence and magnitude of fibrinolytic activation in trauma patients. J Thromb Haemost (2013) 11(2):307–14. 10.1111/jth.12078 23176206

[76] Nielsen VG. A comparison of the thrombelastograph and the ROTEM. Blood Coagul Fibrinolysis (2007) 18(3):247–52. 10.1097/MBC.0b013e328092ee05 17413761

[77] Innerhofer P Westermann I Tauber H Breitkopf R Fries D Kastenberger T The exclusive use of coagulation factor concentrates enables reversal of coagulopathy and decreases transfusion rates in patients with major blunt trauma. Injury (2013) 44(2):209–16. 10.1016/j.injury.2012.08.047 23000050

[78] Tomori T Hupalo D Teranishi K Michaud S Hammett M Freilich D Evaluation of coagulation stages of hemorrhaged swine: comparison of thromboelastography and rotational elastometry. Blood Coagul Fibrinolysis (2010) 21(1):20–7. 10.1097/MBC.0b013e32833113e9 20010092

[79] Zehtabchi S Nishijima DK. Impact of transfusion of fresh-frozen plasma and packed red blood cells in a 1:1 ratio on survival of emergency department patients with severe trauma. Acad Emerg Med (2009) 16(5):371–8. 10.1111/j.1553-2712.2009.00386.x 19302364

[80] Holcomb JB Tilley BC Baraniuk S Fox EE Wade CE Podbielski JM Transfusion of plasma, platelets, and red blood cells in a 1:1:1 vs a 1:1:2 ratio and mortality in patients with severe trauma: the PROPPR randomized clinical trial. JAMA (2015) 313(5):471–82. 10.1001/jama.2015.12 25647203 PMC4374744

[81] Lo CK Mertz D Loeb M. Newcastle-Ottawa scale: comparing reviewers’ to authors’ assessments. BMC Med Res Methodol (2014) 14:45. 10.1186/1471-2288-14-45 24690082 PMC4021422

[82] Hartling L Milne A Hamm MP Vandermeer B Ansari M Tsertsvadze A Testing the Newcastle Ottawa scale showed low reliability between individual reviewers. J Clin Epidemiol (2013) 66(9):982–93. 10.1016/j.jclinepi.2013.03.003 23683848

[83] Higgins JPT Green S. (editors). Cochrane Handbook for Systematic Reviews of Interventions Version 5.1.0 [Updated March 2011]. The Cochrane Collaboration (2011). Available from: http://www.cochrane-handbook.org

