# Supplementary material for: Risk Factors for Trauma-Induced Coagulopathy- and Transfusion-Associated Multiple Organ Failure in Severely Injured Trauma Patients
Source: Front Med (Lausanne). 2015 Apr 24;2:24. doi: 10.3389/fmed.2015.00024 (PMC4408845; doi:10.3389/fmed.2015.00024)
Supplement: Supplementary file 1 [file Data_Sheet_1.PDF]

Supplementary file

Table 1 Quality assessment

Reference	Study design	N	Newcastle-Ottawa Scale		Additional information	Hierarchy of evidence	Delphi score
			**Elements**	**Score**			
Hauser et al. 2010	RCT	573	n.a.	n.a.	n.a.	II	9/10
Boffard et al 2009	RCT	301	n.a.	n.a.	n.a.	II	8/10
Bulger et al. 2007	RCT	209	n.a.	n.a.	n.a.	II	9/10
Waydhas et al. 1998	RCT	40	n.a.	n.a.	n.a.	II	8/10
Trentzsch et al. 2014	Retrospective cohort study	20288	Selection**** Comparability* Outcome***	8/9	Patients from trauma registry of the DGU (German Society for Trauma Surgery)	III	n.a.
Cole et al. 2013	Prospective cohort study	158	Selection**** Comparability Outcome***	7/9	A two-year single center cohort study	III	n.a.
Innerhofer et al. 2013	Prospective cohort study	144	Selection**** Comparability* Outcome***	8/9	A three-year single center cohort study	III	n.a.
Minei et al. 2012	Prospective cohort study	916	Selection**** Comparability** Outcome***	9/9	A multicenter cohort study	III	n.a.
Neal et al. 2012	Prospective cohort study	452	Selection**** Comparability** Outcome***	9/9	A multicenter cohort study	III	n.a.
Kutcher et al. 2012	Prospective cohort study	132	Selection**** Comparability Outcome***	7/9	A three-year single center cohort study	III	n.a.
Duchesne et al. 2012	Retrospective cohort study	188	Selection**** Comparability Outcome***	7/9	A four-year retrospective multicenter study	III	n.a.
Brown et al. 2012	Prospective cohort study	1877	Selection**** Comparability Outcome***	7/9	Multicenter prospective cohort study	III	n.a.
Cohen et al. 2012	Prospective cohort study	203	Selection**** Comparability* Outcome***	8/9	Single center study	III	n.a.
Wafaisade et al. 2011	Retrospective cohort study	1362	Selection*** Comparability* Outcome***	7/9	Multicenter trauma registry of the German Trauma Society	III	n.a.
Perkins et al. 2011	Retrospective cohort study	369	Selection*** Comparability*	8//9	Retrospective review of casualties at the military hospital in Baghdad, Iraq	III	n.a.

			Outcome***				
Nydam et al. 2011	Retrospective cohort study	1415	Selection****<br>Comparability*<br>Outcome***	8/9	A twelve-year single center cohort study	III	n.a.
Hussmann et al. 2011	Retrospective cohort study	375	Selection****<br>Comparability<br>Outcome***	7/9	Patients from trauma registry of the DGU (German Society for Trauma Surgery)	III	n.a.
Nienaber et al. 2011	Retrospective cohort study	36	Selection****<br>Comparability<br>Outcome***	7/9	Patients from trauma registry of the DGU (German Society for Trauma Surgery)	III	n.a.
Brakenridge et al. 2011	Prospective cohort study	1366	Selection***<br>Comparability*<br>Outcome***	7/9	Secondary analysis of a large multicenter prospective observational cohort study	III	n.a.
Borgman et al. 2011	Retrospective cohort study	2474	Selection****<br>Comparability*<br>Outcome***	8/9	Multicenter retrospective study from the Trauma Registry of the German Trauma Society	III	n.a.
Paffrath et al. 2010	Retrospective cohort study	7937	Selection****<br>Comparability<br>Outcome***	7/9	Patients from trauma registry of the DGU (German Society for Trauma Surgery)	III	n.a.
Johson et al. 2010	Prospective cohort study	1440	Selection****<br>Comparability*<br>Outcome***	8/9	Single center study	III	n.a.
Brattstrom et al. 2010	Prospective cohort study	164	Selection****<br>Comparability*<br>Outcome***	8/9	Prospective observational cohort study	III	n.a.
Watson et al. 2009	Prospective cohort study	1175	Selection****<br>Comparability**<br>Outcome***	9/9	Multicenter prospective cohort study	III	n.a.
Mahambrey et al. 2009	Retrospective cohort study	260	Selection***<br>Comparability**<br>Outcome***	8/9	Single center study	III	n.a.
Jastrow et al. 2009	Prospective cohort study	48	Selection****<br>Comparability<br>Outcome***	7/9	Observational nonrandomized single center study	III	n.a.
Englehart et al. 2009	Prospective cohort study	1036	Selection***<br>Comparability<br>Outcome***	6/9	Single center study	III	n.a.
Dewar et al. 2009	Retrospective cohort study	504	Selection****<br>Comparability<br>Outcome***	7/9	Single center study	III	n.a.
Cotton et al. 2009	Prospective cohort study	266	Selection***<br>Comparability*<br>Outcome***	7/9	Single center study	III	n.a.
Maegele et al. 2008	Retrospective cohort study	713	Selection****<br>Comparability*<br>Outcome***	8/9	Patients from trauma registry of the DGU (German Society for Trauma Surgery)	III	n.a.
Sperry et al. 2008	Prospective cohort study	415	Selection****<br>Comparability*	8/9	Multicenter cohort study	III	n.a.

			Outcome***				
Holcomb et al. 2008	Retrospective cohort study	467	Selection****<br>Comparability*<br>Outcome***	8/9	Single center study	III	n.a.
Newell et al. 2007	Retrospective cohort study	1751	Selection****<br>Comparability<br>Outcome***	7/9	Single center study	III	n.a.
Maegele et al. 2007	Retrospective cohort study	8724	Selection****<br>Comparability<br>Outcome***	7/9	Patients from trauma registry of the DGU (German Society for Trauma Surgery)	III	n.a.
Frink et al. 2007	Prospective cohort study	143	Selection****<br>Comparability<br>Outcome***	7/9	A five-year single center cohort study	III	n.a.
Ciesla et al. 2005	Prospective cohort study	1344	Selection****<br>Comparability*<br>Outcome***	8/9	A 12-year inception cohort study	III	n.a.
Raeburn et al. 2001	Prospective cohort study	77	Selection****<br>Comparability<br>Outcome***	7/9	A 4.5-year single center cohort study	III	n.a.
Zallen et al. 1999	Prospective cohort study	63	Selection****<br>Comparability*<br>Outcome***	8/9	Single center study	III	n.a.
Gando et al. 1999	Prospective cohort study	136	Selection****<br>Comparability<br>Outcome**	6/9	Single center study	III	n.a.
Cryer et al. 1999	Prospective cohort study	105	Selection****<br>Comparability*<br>Outcome***	8/9	Single center study	III	n.a.
Sauaia et al. 1998	Retrospective cohort study	411	Selection****<br>Comparability**<br>Outcome***	9/9	Single center study	III	n.a.
Moore et al. 1997	Prospective cohort study	513	Selection****<br>Comparability*<br>Outcome***	8/9	Single center study	III	n.a.
Lehmann et al. 1995	Retrospective study	1112	Selection****<br>Comparability*<br>Outcome***	8/9	Single center study	III	n.a.
Gando et al. 1995	Prospective study	47	Selection****<br>Comparability<br>Outcome**	6/9	Single center study	III	n.a.
Gando et al. 1995	Prospective case-control study	58	Selection****<br>Comparability<br>Outcome**	6/9	Single center study	IV	n.a.
Sauaia et al. 1994	Retrospective cohort study/<br>Prospective cohort study	394	Selection****<br>Comparability*<br>Outcome***	8/9	A three-year cohort study (first year: retrospective; last two years: prospective).	III	n.a.
Waydhas et al. 1994	Prospective cohort study	133	Selection****<br>Comparability	7/9	Single center study	III	n.a.

			Outcome***				
Sigurdsson et al. 1992	Prospective cohort study	21	Selection****<br>Comparability<br>Outcome***	7/9	A ten-month single center cohort study	III	n.a.
Wudel et al. 1991	Retrospective study	5530	Selection****<br>Comparability<br>Outcome***	7/9	Single center study	III	n.a.
Nuytinck et al. 1986	Prospective cohort study	71	Selection****<br>Comparability<br>Outcome***	7/9	Prospective single-center study	III	n.a.

Table 2 Search strategy PubMed and Embase

Pubmed

1. ("Blood Coagulation Disorders"[Mesh] OR "Blood Coagulation"[Mesh] OR Coagulation[tiab] OR coagulopathy[tiab] OR "Fibrinolysis"[Mesh] OR Fibrinolysis[tiab] OR hypofibrinolysis[tiab] OR hyperfibrinolysis[tiab]) OR
2. (“Blood Transfusion”[Mesh] OR Transfusion[tiab] OR “Transfusion Medicine”[Mesh] OR “Erythrocyte Transfusion”[Mesh] OR “Blood Component Transfusion”[Mesh]) AND
3. ("Multiple Organ Failure"[Mesh] OR multiple organ failure*[tiab] OR MOF[tiab]) AND ("Multiple Trauma"[Mesh] OR multiple trauma[tiab] OR "Wounds and Injuries"[Mesh] OR
4. ("Injury Severity Score"[Mesh] OR Injury Severity Score[tiab] OR ISS[tiab])

Embase

1. exp blood clotting disorder/

2. exp blood clotting/

3. exp fibrinolysis/

4. (coagulation or coagulopathy or fibrinolysis or hypofibrinolysis or hyperfibrinolysis).ti,ab.

5. 1 or 2 or 3 or 4

6. exp Blood transfusion/

7. (transfusion* or erythrocyte* or blood component).ti,ab.

8. 6 or 7

9. exp multiple organ failure/

10. (multiple organ failure* or MOF).ti,ab.

11. 9 or 10

12. multiple trauma/

13. exp injury/

14. exp injury scale/

15. (multiple trauma or injury severity score or ISS).ti,ab.

16. 12 or 13 or 14 or 15

17. 5 and 8 and 11 and 16

18. limit 17 to (dutch or english or german)

19. limit 18 to human
